# A Randomized Double-Blind, Placebo-Controlled Study on Anti-Stress Effects of Nelumbinis Semen

**DOI:** 10.3390/ijerph19137963

**Published:** 2022-06-29

**Authors:** Minsook Ye, Hyunsu Bae, Songyi Park, Jaehwan Lew, Kyung Soo Kim, Insop Shim

**Affiliations:** 1 Department of Biomedicine& Health Sciences, College of Medicine, The Catholic University of Korea, Seoul 06591, Korea; jh486ms22@naver.com; 2 Department of Physiology, College of Korean Medicine, Kyung Hee University, Seoul 02447, Korea; hbae@khu.ac.kr; 3 Department of East-West Medicine, Graduate School of East-West Medical Science, Kyung Hee University, Yongin-si 17104, Korea; ssong0111@hanmail.net (S.P.); intmed@khu.ac.kr (J.L.); 4 Department of Family Medicine, College of Medicine, The Catholic University of Korea, Seoul 06591, Korea; 5 Department of Physiology, College of Medicine, Kyung Hee University, Seoul 02447, Korea

**Keywords:** nelumbinis semen (NS), depressive disorder, Beck depression inventory (BDI), electroencephalography (EEG), stress response inventory (SRI)

## Abstract

**Introduction:** Depression is a serious and common mental disease that causes low mood and loss of interest in activities. Nelumbinis semen (NS) has been widely used as a treatment for depression for hundreds of years in many Asian countries. Water extract of nelumbinis semen (WNS) is a standardized herbal medicine made from NS. **Methods:** The objective of the present research was to perform a randomized, double-blind, placebo-controlled trial to estimate the efficacy of WNS for improving depressive and stress symptoms using Beck depression inventory (BDI) and the stress response inventory (SRI) in 45 adults diagnosed with major depression or other forms of depressive disorders. They were randomized to either a placebo-treated group, a 2.4 g per day WNS-treated group, or a 4.8 g per day WNS-treated group. BDI and SRI were determined in order to evaluate changes in depression before and after two weeks of WNS treatment. **Results:** The average BDI and SRI of the 2.4 g WNS-treated group were significantly (*p* < 0.05) improved compared to those of the placebo-treated group. Their BDI subscale A (negative attitudes towards self) and subscale C (somatic disturbances), SRI E, and depression subscale of SRI were substantially shorter (*p* < 0.05). In addition, an analysis of collected EEG data of participants showed a significant increase in alpha/beta activity in the 4.8 g WNS-treated group, which might be explained as an advancement of their depression symptoms (*p* < 0.05). **Conclusions:** These results suggest that WNS treatment can decrease depression. Our study provides preliminary evidence for the safety of WNS and its potential to decrease depression.

## 1. Introduction

Depression is a common mood disease characterized by a low mood with loss of interest or pleasure in usual activities [1]. Depression contributes to global disease burden. The development of antidepressant medications for treating depression has shifted toward serotonin as a key target [2]. Nevertheless, serotonin is not the only factor that plays a role in depression. Based on new research, depression can occur due to various factors such as increased stress hormones, abnormal activities in certain parts of the brain or the immune system, and inflammation. [3]. Accordingly, identifying effective and safe treatments with clear pharmacological mechanisms is urgently needed. Many antidepressant medications are tolerated but side effects suggest that antidepressants are not completely effective [4]. Thus, better antidepressants with greater efficacy and fewer side effects are needed. Although the etiology of depression is unknown, numerous systematic reviews and research papers have been published about the effects of herbal medicine on depression [5].

The Beck depression inventory (BDI), created by Aaron T. Beck in 1998, has become one of the most widely used psychometric methods for detecting depression in normal populations and in different psychiatric patient cohorts which confirm the validity of the Beck depression inventory. We used the Beck depression inventory (BDI), a 21-question multiple-choice self-report inventory, as one of the most widely used psychometric tests for measuring the severity of depression. Its development marked a shift among mental health professionals who had viewed depression from a psychodynamic perspective without considering a patient’s own thoughts.

We used the stress response inventory (SRI), commonly used in clinical practice and psychiatric research [6]. The SRI was devised to score mental and physical symptoms that occurred during the preceding two weeks which might have influenced the status of mental stress level. The SRI includes 39 items focusing on emotional, somatic, cognitive, and behavioral stress responses. SRI scores can be categorized into seven stress factors: tension, aggression, somatization, anger, depression, fatigue, and frustration [6].

Studies of the antidepressant effects of physical exercise based on neurophysiological responses to such exercise activity are scarce. Electroencephalography (EEG) is a reliable tool reflecting upper cognitive functions and mental or psychological states. It is generally considered that higher spectral activity is correlated with arousal, cognitive processing, or emotional activity [7]. Electrical brain activity measured with EEG shows a desynchronized pattern during stress, with strong excited emotions and beta frequencies being dominant. In a relaxed, non-stressful state, EEG contains an alpha activity [8]. Although mechanisms underlying EEG generation are not fully understood, frontal and prefrontal cortices are assumed to play a key role in various rhythmical EEG activities [9]. EEG brainwaves are divided into four bands: delta, theta, alpha and beta. The power spectrum of the beta waves over the alpha waves has a correlation with the level of stress, and the numerical equation of the ratio is given to indicate the state of the subject directly [10].

Nelumbinis semen (NS) has been widely used in Korean traditional medicine to treat insomnia, anxiety, and postmenopausal depression. The use of NS for depressive mood has been reported previously. A previous study has found that NS can exert an antidepressant-like effect in rats, based on a forced swim test. The preclinical study suggested that NS could advance local dopaminergic and cholinergic or norephinergic neurotransmission via activation of cAMP formation in the cortex and hippocampus [11]. Recent studies have found that NS has an antidepressant effect on forced swim-induced depression-like symptoms [12] and chronic mild stress (CMS)-induced depression-like symptoms [11] in rats. These results indicate that NS could serve as an alternative medication to treat persons suffering from depression.

Therefore, this study will extend the appraising process of WNS’s efficacy and safety in improving depression, anxiety, and EEG asymmetry in adults diagnosed with depression through a randomized, double-blind, placebo-controlled clinical trial. Furthermore, we examined the ratio of α/β activity using EEG to provide an assessment of stress. 

## 2. Methods

### 2.1. Study Design and Participants

In this study, 46 participants were randomly recruited. Permission to perform the study was granted by the Ethical Review Board of Human Research of the College of Medicine at Catholic University (Seoul, Korea) after ethical approval by its ethical committee, with code number KCMC07MS274. Each participant submitted prior written consent. This study was designed as a double-blind, placebo-controlled, randomized clinical trial conducted in South Korea. All participants were Koreans. Participants were aged 18–65 years. All participants were screened for BDI. They were divided into three groups (a placebo-treated group, a 2.4 g WNS-treated group, and a 4.8 g WNS-treated group) based on computer-generated random selections.

Criteria for patient inclusion in this study were: those who were ready and capable of conforming with the study protocol, those who were capable of understanding and selecting Korean and English alphabets, those who were capable of communicating with clinical trial performers fully, those who were capable of complying with all tests and medical examinations demanded by the study protocol, those who were capable of sufficiently understanding the study purpose, and those who were willing to indicate consent by adding their names or seal. Exclusion criteria were: those who had a fasting glucose level of over 126 mg/dl, systolic blood pressure of over 140 mm Hg or diastolic blood pressure of 90 mm Hg, blood thyroid-stimulating hormone below 0.3 or over 4.0, hemoglobin below 13.0 g/dl for men or 12.0 g/dl for women, abnormal liver function exceeding 1.5 times normal levels of ALT or AST, infections in upper airways or chronic diseases, as well as mentally ill patients.

### 2.2. Study Medications

We used hard gelatin capsules containing 400 mg of WNS. A dry extract of the herbal medicine was manufactured by Sun Ten pharmaceutical company in Taiwan. Participants received treatment (12 capsules per day taken twice daily) for 2.4 g of WNS or 4.8 g of WNS, or a placebo, for two weeks. Subjects in the placebo group received capsules containing an equivalent amount of starch and lactose.

Results from participants experiencing 11 effective days (80% of 14 days) of treatment were used for the final analysis, i.e., 11 days after taking the capsules. Patients with protocol violations were excluded from the final analysis. Study drugs were labeled sequentially by the manufacturing department of the sponsor based on a randomization list. They were provided to investigators in blocks of three. All investigators and personnel who were actively involved in this trial were blinded to group assignment until the database was closed. After completion of this trial, drug supplies that were not used were returned to the monitor.

### 2.3. Measures

#### 2.3.1. Beck Depression Inventory (BDI)

BDI is a 21-item self-rated inventory. Each item was rated with a set of four possible answer choices of increasing intensity. When the test was scored, a value of 0 to 3 was assigned for each answer and then the total score was compared to a key to determine the depression’s severity. BDI had three subscales: (1) negative attitudes towards self (A), (2) performance impairment (B), and (3) somatic disturbances (C). The highest score on each of these 21 questions was 3 and the highest possible total score for the whole test was 63. The lowest possible score for the whole test was zero. Only one score per question (the highest rated if more than one was circled) was added. An index score ranging from 5 to 9 was considered to be within the normal range. A score of 10 to 15 indicated mild depression. A score of 16–23 meant moderate depression and a score of 24–63 indicated severe depression. A score below 4 was recognized as possible denial of depression or faking good health. Thus, participants who received scores below 4 were excluded.

#### 2.3.2. Stress Response Inventory

The SRI scale consisted of a total of 39 response items under seven subscales (tension, aggression, somatization, anger, depression, fatigue, and frustration). When the test was scored, a value of 0 to 4 was assigned for each answer. The total score was then compared to a key to determine severity of the stress.

### 2.4. Electroencephalogram Recording and Analyses

Brain wave activity was assessed by a trained professional researcher using EEG spectra with a PolyG-1 (LAXTHA, Daejeon, Korea). Electrodes were attached to cortical sites on the participants’ prefrontal lobes (Fp1, Fp2) according to the international 10/20 system. Among several types of EEG waveforms, including α and β waves, only the α and β waves were measured since they were key indicators of brain arousal and relaxation. EEG measurement time varied depending on the purpose of the study and characteristics of participants. EEG data were quantitatively analyzed using Telescan 2.98 (Laxtha Inc., Daejeon, Korea). Sixty seconds of raw EEG data were analyzed for each measurement (after excluding the first and last 10 s of recording). In addition, only EEG waves between 5 and 50 Hz were analyzed. To correct EEG differences between subjects due to variations in scalp thickness and tension levels, a relative band power was calculated from the absolute band power using the ratio of the absolute band power at a certain frequency. Relative alpha power (10–13 Hz) and relative beta power (20–30 Hz) were then analyzed.

### 2.5. Statistical Analysis

Data were statistically analyzed using one-way analysis of variance (ANOVA) followed by an LSD post-hoc test. Statistical significance in the same subject group was determined using a paired t-test, and the gender comparison was determined using a chi-square test.

All of the results are presented as mean ± standard error of mean (SEM). IBM SPSS Statistics 23.0 for Windows was used for analysis of the statistics. Statistical significance was set at *p* < 0.05.

## 3. Results

### 3.1. Participants’ Characteristics at Entry

**Figure 1:**
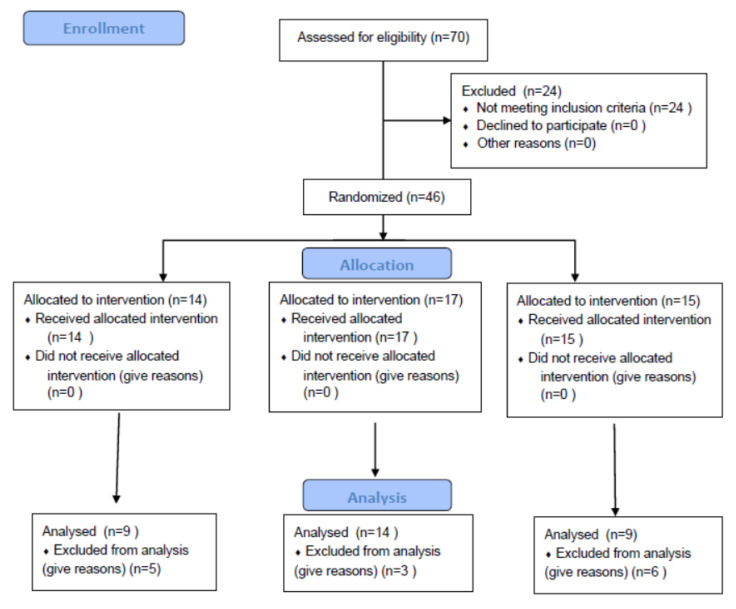
The study flowchart.

A total of 70 people participated in this trial with their permission at the start. After applying inclusion and exclusion criteria, 58 volunteers were found to be eligible for the study. These 58 volunteers underwent a BDI test. Of them, 46 had BDI score above 5. Finally, these 46 volunteers were randomly assigned to treatment groups of 2.4 g WNS (14 cases), 4.8 g WNS (17 cases), and placebo (15 cases). The study flowchart is shown in Figure 1. These three groups did not differ in age or sex distribution after randomization. All 46 volunteers initially randomized completed this study. However, for the final analysis, only results from participants experiencing 11 effective days (80% of 14 days) were used. Based on this criterion, five volunteers in the placebo group, three volunteers in the 2.4 g WNS group, and six volunteers in the 4.8 g WNS-treated group were excluded. Thus, results of the remaining 32 volunteers were finally analyzed. Table 1 shows characteristics of these subjects.

### 3.2. WNS’s Effect on BDI and SRI Scores

**Figure 2:**
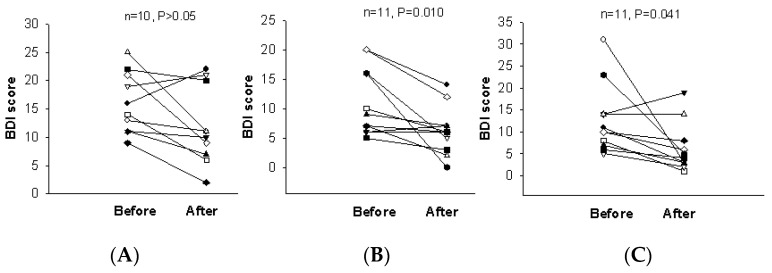
Differences in BDI mean scores between WNS and placebo groups. (**A**) Placebo group, (**B**) 2.4 g of WNS group, (**C**) 4.8 g of WNS group.

**Figure 3:**
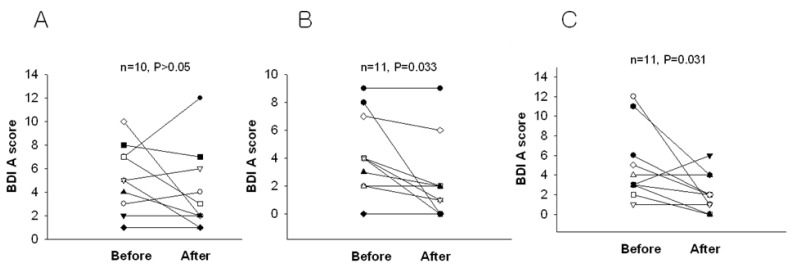
Differences in BDI subscale A mean scores of WNS and placebo groups. (**A**) Placebo group, (**B**) 2.4 g of WNS group, (**C**) 4.8 g of WNS group.

**Figure 4:**
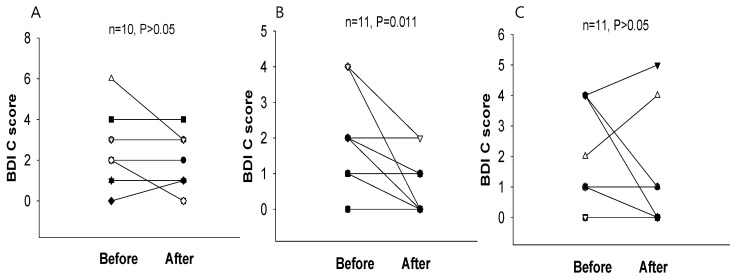
Differences in BDI subscale C mean scores between WNS and placebo groups. (**A**) Placebo group, (**B**) 2.4 g of WNS group, (**C**) 4.8 g of WNS.

**Figure 5:**
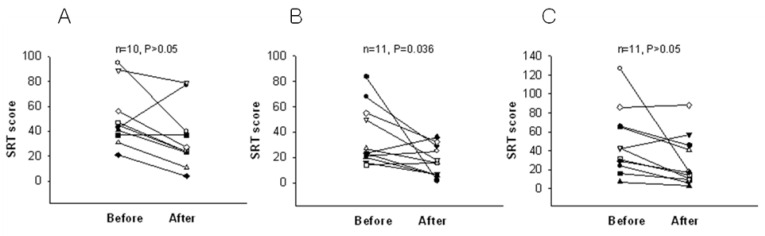
Differences in SRI mean scores between WNS and placebo groups. (**A**) Placebo group, (**B**) 2.4 g of WNS group, (**C**) 4.8 g of WNS group.

**Figure 6:**
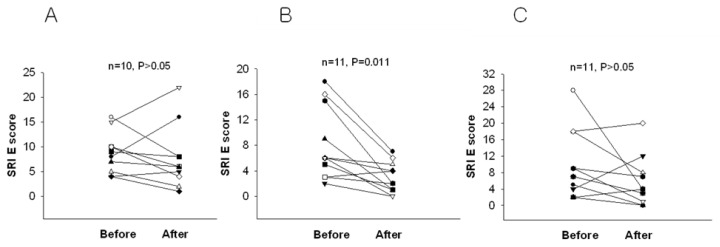
Differences in SRI E mean scores between WNS and placebo groups. (**A**) Placebo group, (**B**) 2.4 g of WNS group, (**C**) 4.8 g of WNS group.

When the test was scored, participants who received scores below 4 were excluded since these scores are below usual scores for normal, recognized as possible denial of depression or faking good health. After two weeks of intervention, the BDI score was significantly reduced in the 2.4 g and 4.8 g WNS-treated groups as compared with that in the placebo group (*p* < 0.05) (Table 2 and Figure 2). BDI subscale A (negative attitudes towards self) was substantially shorter in the 2.4 g and 4.8 g WNS-treated group (*p* < 0.05) than that in the placebo group (Figure 3). BDI subscale C showed a trend toward a shorter decrease in the WNS 2.4 g group than that in the placebo group (*p* < 0.05) (Figure 4). SRI mean scores were significantly reduced in the WNS 2.4 g treatment group (*p* < 0.05) (Table 3 and Figure 5 and Figure 6). Paired t-tests revealed that SRI E was substantially shorter in the 2.4 g WNS-treated group (*p* < 0.05). These results suggest that WNS 2.4 g treatment can reduce depression. Thus, WNS treatment may help depressed patients recover from depression in clinics.

### 3.3. Resting EEG Asymmetry

**Figure 7:**
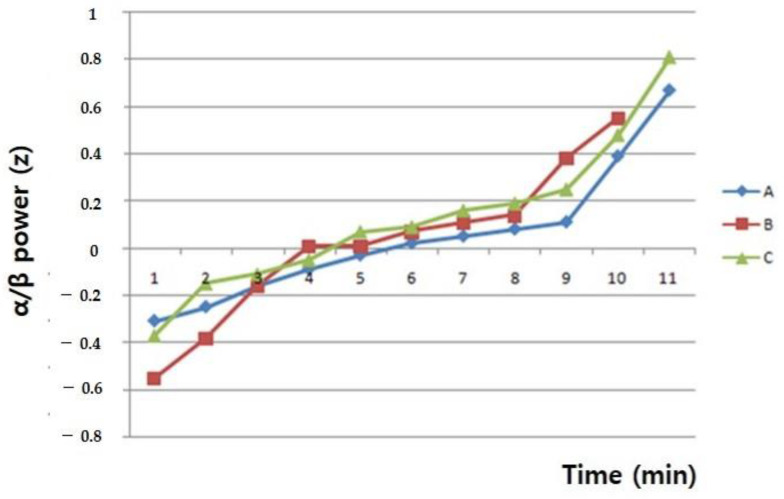
Asymmetry values with depressive disorder after WNS and placebo treatment. (A) 2.4 g of WNS group, (B) placebo group, (C) 4.8 g of WNS.

The analysis carried out on EEG power spectral content showed differential effects for each EEG band with marked topographical differences. As depicted in Figure 7, the alpha–beta ratio was significantly increased in both WNS 2.4 g and 4.8 g treatment groups.

## 4. Discussion

Depression is a common and serious mood disorder that is a worldwide problem for humans because of its significant association with disorders such as sleep disturbances, low self-esteem, guilty feelings, and suicidal tendencies [12]. However, depression is considered a complex disorder. Mechanisms underlying its pathogenesis remain unclear. Existing treatments for depression are unsatisfactory. Searching for an effective treatment for patients suffering from depression is a major challenge. The aim of this study was to verify antidepressant effects of NS on symptoms of depression and changes caused on EEG frontal asymmetry.

Nelumbinis semen has been recognized as a traditional natural medicine as a remedy. Although NS effects and mechanisms are still largely unknown, NS has been used to cure diverse mental diseases in traditional medicine. Several studies have reported the pharmacological efficacy of NS in various disease models. A recent research study has suggested that NS possesses pharmacological effects and various activities, such as hypoglycemic, antidiarrheal, antimicrobial, diuretic, antipyretic, anti-inflammatory, hepatoprotective, anti-proliferative, and antioxidant activities. Several studies have also reported anti-depression effects of NS in various depressant animal models. In previous studies, we have found that NS has a clear antidepressant effect in that it can reduce the immobility time of rats in forced swim tests [11] and reverse decreases of sucrose intake and serotonin (5-HT)1A receptor binding in the hippocampus (5-HT1A hetero-receptors) induced by CMS. NS contains diverse kinds of alkaloids [13]. Among these alkaloids, neferine is known for its anti-arrhythmic actions [14] and tranquilization action [15], while isoquercetin is known to possess spasmolytic action [16]. In particular, anonaine, asimilobine, isoquercetin, hyperoside, lirinidine, and nornuciferine elements of NS are known to have anti-depression effects as they can increase 5-HT concentration by neurotransmitter reuptake inhibition and increase 5-HT receptor-binding potential [17,18,19,20]. These effects might be mediated by components in NS such as serotonin receptor agonists anonaine and asimilobine [21]. Our previous studies have reported that NS might possess an antidepressant effect by enhancing serotonin level.

Previous work has also suggested that quantitative sleep EEG can predict clinical responses to antidepressants [22]. However, whether underlying mechanisms of these states could be applied to EEG signals remains unclear. Some researchers have suggested a need to pay more attention to the prefrontal cortex [23]. A previous study has stated that the EEG of a person under stress displays a decrease in alpha (11–12 Hz) activity and an increase in EEG amplitude in 19–22 Hz and high beta (23–35 Hz) ranges [24]. The present study showed that after intervention with NS, the ratio of α/β activity was increased by 51% in the 4.8 g treatment group and 26% in the 2.4 g treatment group compared to that in the placebo group. Our findings provide evidence for the assumed antidepressant effect of NS by changing the ratio of α/β activity in EEGs of the frontal cortex.

BDI and SRI are appropriate and convincing evaluations used to detect many symptoms of depression and anxiety, respectively [25]. Both study groups showed enhanced scores from baseline using both assessment tools. Taking WNS treatment reduced BDI and SRI mean scores in these patients. To the best of our knowledge, this is the first study to report the effect of WNS treatment on clinical status of patients with depression. The 2.4 g WNS group showed greater improvements based on SRI. Our results showed that WNS significantly reduced BDI and SRI mean scores in depressed patients, suggesting that decreases in BDI and SRI mean scores might be related to the therapeutic effect of WNS. It should be noted that treatment with WNS 2.4 g resulted in pronounced improvement of BDI and SRI scores and tended to be slightly larger than that produced by WNS 4.8 g. The exact reasons for these effects were unknown. However, efficacy outcomes from this BDI or SRI suggest that there may be a dose ceiling effect at 2.4 g and this dose may produce a saturating level for depressive symptoms while good drug effects are observed at 2.4 g. There is a need for further research on the use of lower dosages such as 0.6 or 1.2 g/kg in order to find out the most effective and appropriate dosage in the future.

In previous studies, we found that the most effective dose of NS was 400 mg/kg among 100, 200, 400, or 1000 mg/kg of NS in animal studies [18,26]. Based on these animal studies, 2.4 g and 4.8 g/kg were selected in the present study.

Although our study has some limitations, this is the first study that evaluated the association between NS and depression. In addition, assessment of the ratio of α/β activity in the EEG could be used for the estimation of depressive symptoms. In addition, the molecular mechanism underlying the antidepressant effect of WNS requires further study before firm conclusions can be drawn. In summary, treatment with WNS can attenuate depression in depressive patients.

## 5. Conclusions

In conclusion, the anti-stress effects of WNS are associated with reduced mean scores of BDI and SRI and increased ratios of α/β activity in the EEG of patients with depression. These results support the development of a novel antidepressant established on the pharmacological action of nelumbinis semen. Based on the results of the present study, we conclude that WNS has potential for treating depression.

## Figures and Tables

**Table 1:** Characteristics of randomized subjects.

	Placebo	WNS 2.4	WNS 4.8	*p*-Value	χ^2^
	(n = 15)	(n = 14)	(n = 17)		
Age (years)	35.5 ± 3.2	31.7 ± 3.1	36.1 ± 2.4	0.367	-
Male–female ratio	8/7	5/9	6/11	1	1.904

Values are presented as mean ± SEM; *p*-values were calculated using **χ^2^** test unless otherwise specified.

**Table 2:** Changes in total BDI scores and BDI subscale scores in patients after WNS treatment for two weeks.

		Placebo	WNS 2.4	WNS 4.8
		(n = 10)	(n = 11)	(n = 11)
		Values	Values	Values
BDI	Before	16.1 ± 1.7	11.1 ± 1.7	12.6 ± 2.4
	After	11.9 ± 2.2	6.2 ± 1.2 ^a^	6.2 ± 1.7 ^b^
	difference	4.2 ± 1.9	4.9 ± 1.5	6.5 ± 2.8
BDI A	Before	5.2 ± 0.9	3.9 ± 0.9	5.0 ± 1.1
	After	4.0 ± 1.1	2.1 ± 0.9 ^c^	2.2 ± 0.6 ^d^
	difference	1.2 ± 1.1	1.8 ± 0.7	2.8 ± 1.1
BDI B	Before	8.5 ± 0.7	6.6 ± 0.8	6.6 ± 0.6
	After	6.2 ± 0.9 ^e^	4.5 ± 0.7 ^f^	4.2 ± 0.7 ^g^
	difference	2.3 ± 0.9	2.1 ± 0.8	2.5 ± 1.0
BDI C	Before	2.4 ± 0.5	1.7 ± 0.4	1.7 ± 0.5
	After	1.8 ± 0.4	0.5 ± 0.2 ^h^	1.1 ± 0.5
	difference	0.6 ± 0.4	1.2 ± 0.4	0.6 ± 0.5

Values are ±SEM; ^a^ *p* = 0.033, paired *t*-test; ^b^ *p* = 0.041, paired t-test; ^c^ *p* = 0.033, paired t-test; ^d^ *p* = 0.031, paired t-test; ^e^ *p* = 0.036, paired t-test; ^f^ *p* = 0.030, paired t-test; ^g^ *p* = 0.029, paired t-test; ^h^ *p* = 0.011, paired t-test.

**Table 3:** Changes in total SRI scores and SRI subscale scores in patients after WNS treatment for two weeks.

		Placebo	WNS 2.4	WNS 4.8
		(n = 10)	(n = 11)	(n = 11)
		Values	Values	Values
SRI	Before	50.5 ± 7.5	36.5 ± 7.2	48.7 ± 10.6
	After	34.6 ± 8.0	17.5 ± 3.5 ^a^	28.1 ± 8.1
	difference	15.9 ± 7.1	18.9 ± 7.8	20.6 ± 9.7
SRI A	Before	8.1 ± 1.2	4.3 ± 1.2	6.4 ± 1.5
	After	4.5 ± 1.2 ^b^	1.5 ± 0.4	3.5 ± 1.2
	difference	3.6 ± 1.5	2.7 ± 1.3	2.9 ± 1.3
SRI B	Before	3.0 ± 1.0	2.3 ± 0.9	3.2 ± 1.4
	After	2.0 ± 0.9	0.9 ± 0.6	1.9 ± 0.8
	difference	1.0 ± 0.9	1.4 ± 1.0	1.3 ± 1.3
SRI C	Before	3.9 ± 1.0	2.7 ± 0.8	3.1 ± 0.7
	After	1.7 ± 0.5	1.0 ± 0.4 ^c^	2.1 ± 0.6
	difference	2.2 ± 1.1	1.7 ± 0.8	1.0 ± 0.5
SRI D	Before	7.8 ± 1.1	5.4 ± 0.9	8.4 ± 2.1
	After	6.3 ± 1.3	3.5 ± 0.9	4.9 ± 1.6
	difference	1.5 ± 1.1	1.9 ± 1.3	3.5 ± 1.6
SRI E	Before	8.8 ± 1.3	8.1 ± 1.7	9.9 ± 2.5
	After	7.8 ± 2.0	2.9 ± 0.7 ^d^	5.6 ± 1.8
	difference	1.0 ± 1.6	5.2 ± 1.4	4.3 ± 2.5
SRI F	Before	8.0 ± 1.4	7.2 ± 1.2	7.4 ± 1.4
	After	5.6 ± 1.5 ^e^	3.9 ± 0.9 ^f^	4.9 ± 1.1
	difference	2.4 ± 0.9	3.3 ± 1.5	2.5 ± 1.2
SRI G	Before	11.2 ± 2.5	6.5 ± 1.6	10.5 ± 2.3
	After	6.7 ± 1.6	3.8 ± 0.8	5.2 ± 1.6 ^g^
	difference	4.5 ± 2.0	2.7 ± 1.8	5.3 ± 2.1

Values are presented as mean ± SEM. ^a^ *p* = 0.036, paired t-test; ^b^ *p* = 0.041, paired t-test; ^c^ *p* = 0.044, paired t-test; ^d^ *p* = 0.004, paired t-test; ^e^ *p* = 0.024, paired t-test; ^f^ *p* = 0.048, paired t-test; ^g^ *p* = 0.029, paired t-test.

## Data Availability

Not applicable.
